# Effects of synovial macrophages in osteoarthritis

**DOI:** 10.3389/fimmu.2023.1164137

**Published:** 2023-07-10

**Authors:** Kun Zhao, Jiaqi Ruan, Liuyan Nie, Xiangming Ye, Juebao Li

**Affiliations:** ^1^ Center for Rehabilitation Medicine, Rehabilitation and Sports Medicine Research Institute of Zhejiang Province, Department of Rehabilitation Medicine, Zhejiang Provincial People’s Hospital (Affiliated People’s Hospital, Hangzhou Medical College), Hangzhou, Zhejiang, China; ^2^ Zhejiang University School of Medicine, Hangzhou, Zhejiang, China; ^3^ Department of Rheumatology, Sir Run Run Shaw Hospital, Zhejiang University School of Medicine, Hangzhou, China

**Keywords:** osteoarthritis, macrophages, synovial, polarization, treatment, review

## Abstract

Osteoarthritis (OA) is a common degenerative disease in mammals. However, its pathogenesis remains unclear. Studies indicate that OA is not only an aging process that but also an inflammation-related disease. Synovitis is closely related to the progression of OA, and synovial macrophages are crucial participants in synovitis. Instead of being a homogeneous population, macrophages are polarized into M1 or M2 subtypes in OA synovial tissues. Polarization is highly associated with OA severity. However, the M1/M2 ratio cannot be the only factor in OA prognosis because intermediate stages of macrophages also exist. To better understand the mechanism of this heterogeneous disease, OA subtypes of synovial macrophages classified by gene expression were examined. Synovial macrophages do not act alone; they interact with surrounding cells such as synovial fibroblasts, osteoclasts, chondrocytes, lymphocytes and even adipose cells through a paracrine approach to exacerbate OA. Treatments targeting synovial macrophages and their polarization are effective in relieving pain and protecting cartilage during OA development. In this review, we describe how synovial macrophages and their different polarization states influence the progression of OA. We summarize the current knowledge of the interactions between macrophages and other joint cells and examine the current research on new medications targeting synovial macrophages.

## 1 Introduction

Osteoarthritis (OA) is a degenerative joint disease caused by chronic inflammation in cartilage, subchondral bone and synovium. In osteoarthritic joints, major pathological changes include damage to articular cartilage, sclerosis and cystic degeneration of subchondral bone, and hyperplasia of the synovial membrane and articular capsule. As the most common joint disease, OA causes pain and disability and creates a substantial burden on individuals and society (1). Despite the major public burden posed by OA, effective medicine for its treatment remains insufficient. Current clinical drugs for OA, including local glucocorticoids and nonsteroidal anti-inflammatory drugs (NSAIDs), can suppress the pain caused by OA but cannot stop its progression. Therefore, clarifying the mechanism of OA initiation and discovering useful treatments have become urgent tasks for scientists and researchers.

**Figure 1:**
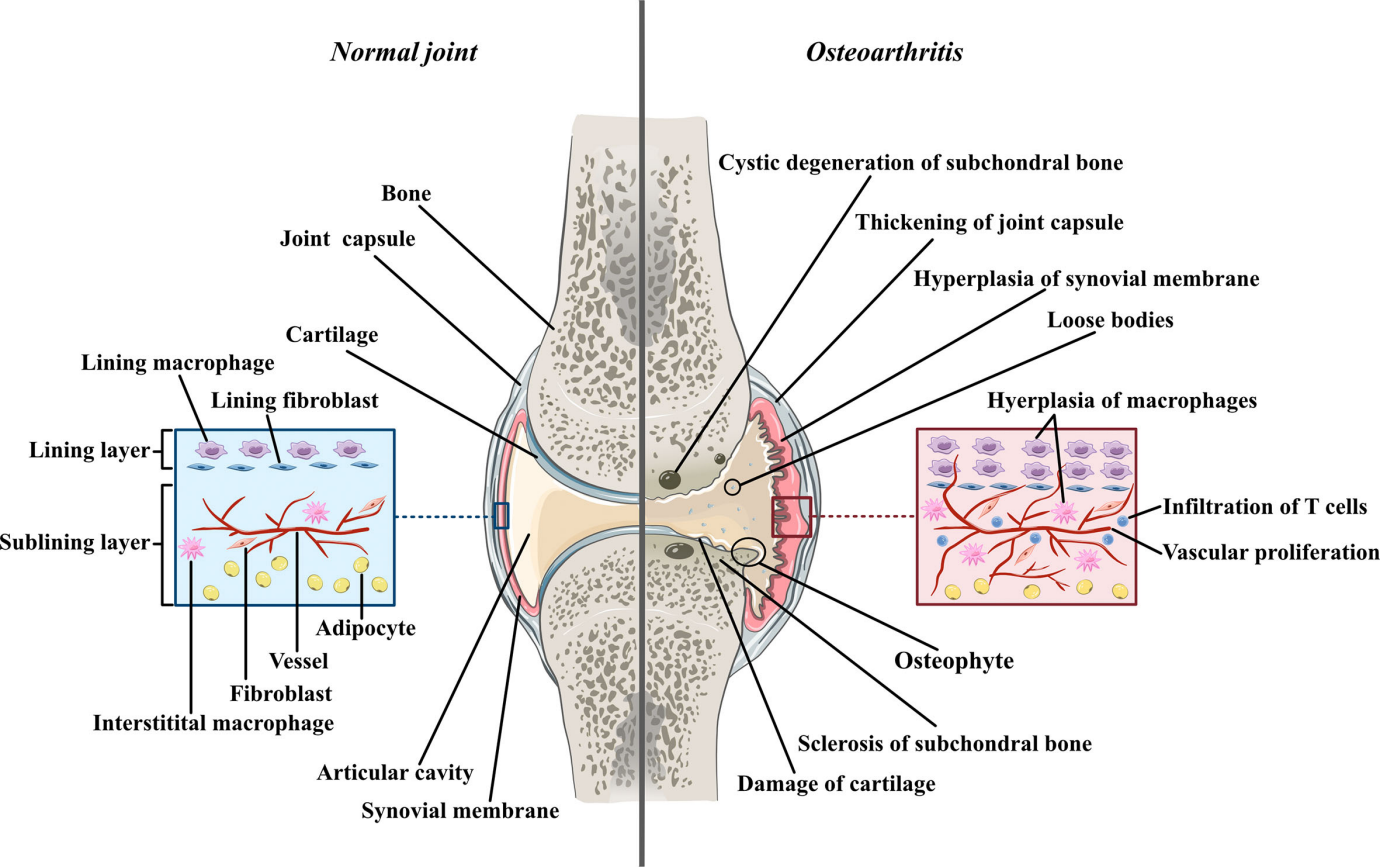
The comparison between normal joint and osteoarthritic joint Normal joint composed of joint cartilage, joint capsule, synovial membrane and articular cavity. Generally, synovial membrane has two layers. The inner layer, also called lining layer is mainly composed of synovial macrophage and synovial fibroblasts. The outer layer of synovial membrane also called sublining layer is connective tissue composed of blood vessels, collagens, interstitial macrophages, fibroblasts, adipocytes and a small number of lymphocytes. In osteoarthritic joint, the articular cartilage is damaged and the fragments of cartilage drift into articular cavity, forming loose bodies. The loss of cartilage leads to unevenly distributed loads on subchondral bones which can further cause osteoproliferation and sclerosis in the friction part as well as bone resorption and cystic degeneration in the periphery part. Moreover, both synovial membrane and articular capsule thicken in osteoarthritic joint due to inflammation. In hyperplastic synovial membrane, the most prominent changes are the hyperplasia of synovial and interstitial macrophages, the infiltration of T lymphocytes in sublining layer and the proliferation of blood vessels.

Osteoarthritis was used to be thought as a degenerative disease mostly caused by cartilage defects before accumulating research reveals the significant contribution of synovial inflammation in the progression of OA. Imaging studies coupled with histopathologic analyses have demonstrated that synovitis could facilitate the pathogenesis of OA (2). The normal synovium has two layers (**Figure 1**). The inner layer, which is also called the lining layer, is mainly composed of synovial macrophages and synovial fibroblasts and plays an important role in maintaining joint homeostasis. The outer layer of the synovium consists of different types of connective tissue that assist various joint functions. However, in the OA synovium, the number of lining layer cells, especially the number of synovial macrophages, is increased (3). Microscopically, the appearance of OA can be indistinguishable from that observed in RA. They both involve neovascularization and infiltration by fibroblasts and macrophages. However, their histological changes are different. In contrast to the synovial inflammation observed in rheumatoid arthritis (RA), synovial inflammation in OA is not a diffuse process: its distribution is patchy and confined to areas adjacent to sites of chondropathy (2). The accumulation of macrophages in OA is not caused by a single factor but arises from complicated interactions between joint cells such as chondrocytes, fibroblasts, and lymphocytes. Molecules such as soluble matrix degradation products (SMDPs) from cartilage, fibronectin, adipokines and some alarmins act as danger-associated molecular patterns (DAMPs), which activate macrophages to produce inflammatory cytokines and chemokines through surface pattern recognition receptors (PRRs), such as Toll-like receptors (TLRs), which are expressed on the surface of macrophages. Additionally, cytokines and chemokines such as interleukins (ILs), growth factors and monocyte chemotactic protein (MCP) produced by various cells in the joint also play a role in macrophage activation and accumulation by binding to the corresponding receptors and further activating the JAK-STAT pathway (4). Macrophages play a crucial role in OA synovitis, and their polarization correlates with the progression of OA. Macrophages can be divided into three phenotypes according to their function: unstimulated macrophages (M0), proinflammatory macrophages (M1) and anti-inflammatory macrophages (M2). M0 cells are resting and preactivated macrophages, which can transform into M1 or M2 macrophages under certain microenvironments. M1 macrophages, as an anti-pathogene soldier, can be induced by bacterial lipopolysaccharide (LPS) or Th1 cytokines such as interferon-γ (IFN-γ), TNF-α both *in vitro* and *in vivo.* Activated M1 macrophages could secrete proinflammatory cytokines such as TNF-α and IL-1. In contrast, M2 macrophages are polarized by IL-4 and IL-13 and exert anti-inflammatory effects and tissue repair and remodeling functions (5). These macrophage inductors are still frequently used in *in vitro* experiments to induce M1/M2 and explore their effects in specific diseases, as well as in the main topic of our thesis -osteoarthritis. Despite all the knowledge about macrophage accumulation and polarization, the specific mechanism by which this occurs in OA joints has not been elucidated. Here, we aimed to share the current evidence of the role of synovial macrophages and their subsets in OA progression. We also summarized the current knowledge of interactions between macrophages and other joint cells, such as chondrocytes and fibroblasts. In this review, possible treatments for OA targeting polarized synovial macrophages are also summarized.

## 2 Synovial macrophages are activated and proliferate in OA

### 2.1 *In vitro* experiments

In 1991, the antigen-antibody technique was used to show that normal synovial lining cells expressed many macrophage-associated antigens, such as CD11b, CD16, CD14 and CD68 (6). Haywood L et al. collected synovial tissue samples from 104 OA patients in 2003 and found that angiogenesis and synovial lining thickness increased with increasing macrophage fractional areas (7). Afterward, Benito MJ showed significantly greater CD14^+^ and CD68^+^ cell infiltration, blood vessel formation and intercellular adhesion molecule-1 expression were detected in synovial tissue from OA patients than in normal individuals (3). In 2006, Bondeson J found that fewer proinflammatory cytokines, such as IL-1 and TNF-α, were produced in digested osteoarthritis synovium cultures after depleting CD14^+^ cells (CD14 is expressed by classic mononuclear macrophages) using anti-CD14-conjugated magnetic beads. These results suggested that the number of macrophages was increased in the synovium of OA patients and that these proliferated synovial macrophages could exacerbate OA by producing proinflammatory cytokines such as TNF-α and IL-1. However, *in vitro* studies could not dynamically observe the activation of synovial macrophages in the body. Tracing the changes in synovial macrophages in the body was impossible until the discovery of the radionuclide labeling technique (8).

### 2.2 *In vivo* studies based on radionuclide-labeled folate

According to their distinct molecular phenotypes, monocytes can be divided into two subgroups: the CD14^+^CD16^-^ subgroup and the CD14^-^CD16^+^ subgroup. The first subgroup, which are called classic monocytes, has classic proinflammatory functions such as infiltration and macrophage transformation. The second subgroup is considered to have increased cytokine expression and antigen-presenting abilities (9, 10). It is frequently reported that folate receptor-β (FR-β) is specifically expressed on the surface of the classic CD14^+^CD16^-^ monocyte subgroup and derived macrophages. Therefore, radionuclide-labeled folate is frequently used to detect the quantity and distribution of activated macrophages *in vivo (* 11).

In 2016, Kraus VB provided direct *in vivo* evidence for the connection between synovial macrophages and OA for the first time. Kraus VB injected radionuclide-labeled folate called 99mTc–EC20 (etarfolatide) into twenty-five individuals with symptomatic OA, after which SPECT-CT imaging of both knees and planar imaging of the whole body were used to detect activated macrophages *in vivo*. The results showed that activated macrophages were present in 76% of OA knees, and etarfolatide uptake was mainly detected in the synovium and joint capsule. Radiographic knee OA severity and joint symptoms significantly correlated with synovial and capsular etarfolatide uptake. In addition, whole-body planar imaging suggested that activated macrophages were localized to joints that were susceptible to OA, including hand finger joints, thumb bases, shoulders, great toes and ankles. Pain in these joints was also positively associated with the quantity of activated macrophages (12). These findings indicate that activated macrophages are increased in OA joints and that most of them accumulate in the synovium.

### 2.3 Modulating the quantity of synovial macrophages affects OA

After learning that synovial macrophages are increased in OA patients, researchers have tried to manipulate the quantity of synovial macrophages in animal models to further verify the connection between synovial macrophages and OA.

Clodronate-laden liposomes are toxic to all phenotypes of macrophages, and so they are commonly used to deplete macrophages. In 2004, van Lent PL injected clodronate-laden liposomes into mouse knee joints to selectively deplete synovial lining macrophages. Then, TGF-β was injected into knee joints to induce osteophyte formation. Van Lent PL found that osteophyte formation and the production of bone morphogenetic protein (BMP)-2 and BMP-4 were dramatically reduced after the depletion of synovial lining macrophages (13). Additionally, Takano S discovered that the expression of IL-1β, TNF-α and nerve growth factor (NGF, a cytokine associated with pain) in the synovium was reduced after depleting synovial macrophages in OA mice (14). Using a similar method, many other studies showed that the production of proinflammatory cytokines (IL-1β, TNF-α), the formation of osteophytes, and cartilage destruction were alleviated after selectively depleting synovial macrophages (15, 16). Despite these positive results, Wu CL’s study showed conflicting findings. He found that OA was exacerbated after depleting synovial macrophages from transgenic obese mice with Fas-induced macrophage apoptosis using the small molecule AP20187. The author explained that it was possible that macrophages repopulated after AP20187 administration and that the repopulated macrophages contributed to the progression of OA. Another explanation was that synovial macrophages could protect against ox-LDL-induced inflammation, and so after macrophage depletion, this protection was no longer present (17).

## 3 Macrophage polarization in the OA synovium

### 3.1 Discovery of M1/M2 macrophages in the OA synovium

**Figure 2:**
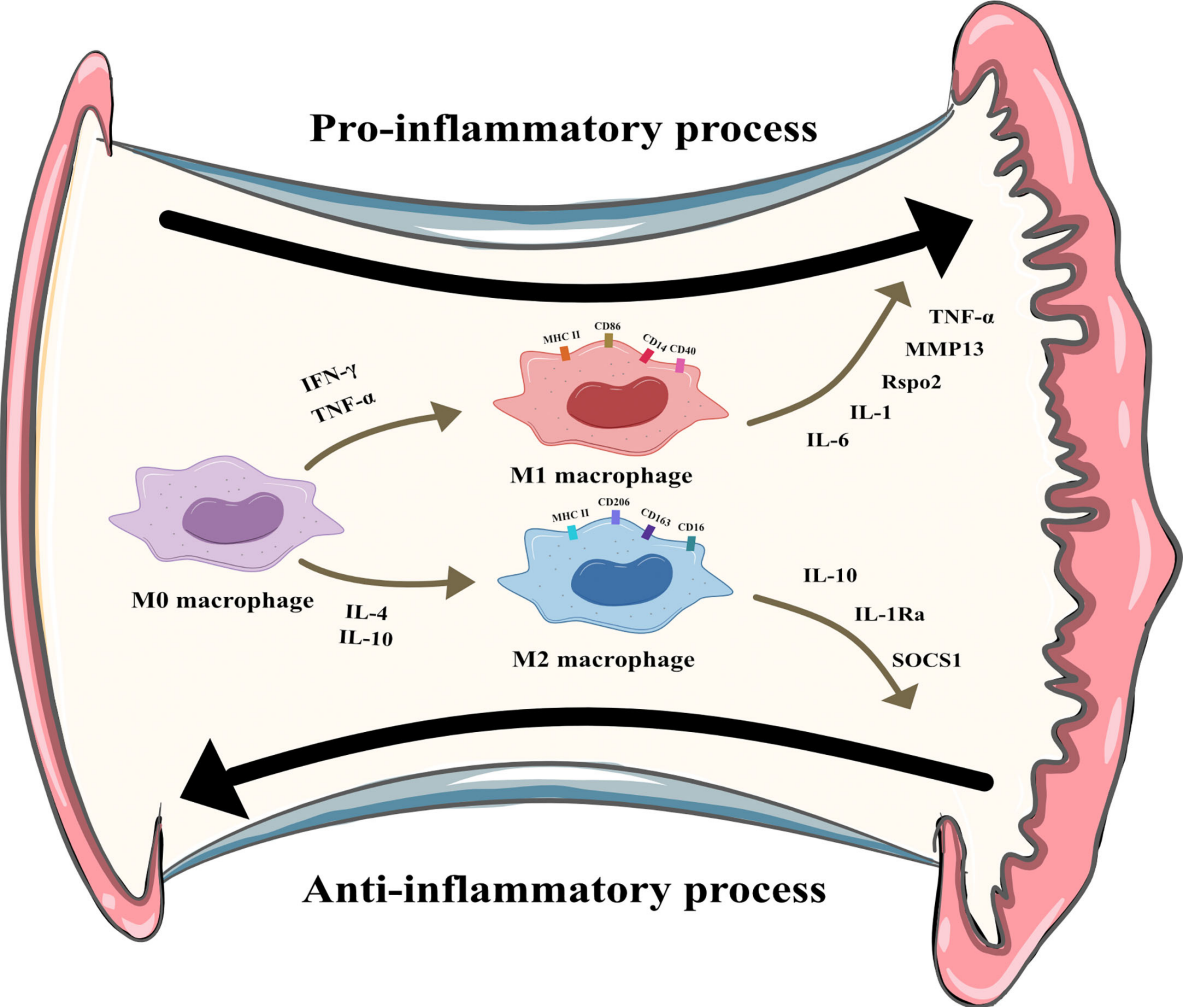
M1 and M2 polarization of synovial macrophages. M1 macrophages can be induced by Interferon-γ (IFN-γ) and tumor necrosis factor-α (TNF-α) and secret pro-inflammatory cytokines like interleukin (IL)-6, IL-1, R-spondin-2 (Rspo2), matrix metalloproteinase-13(MMP13), TNF-α. M2 macrophages can be induced by IL-4 and IL-10 and secret anti-inflammatory cytokines like IL-10, IL-1 receptor antagonist, suppressor of cytokine signaling 1 (SOCS1).

Macrophages can be divided into multiple phenotypes. Among these phenotypes, the two most important subtypes are “classically activated macrophages” (M1 polarization) and “alternatively activated macrophages” (M2 polarization). M1 macrophages can be induced by IFN-γ and TNF-α *in vitro*. Characterized by CD80, CD86, and MHCII expression, these cells are known as proinflammatory macrophages due to their ability to produce proinflammatory cytokines such as TNF-α and IL-1. Moreover, they can release crucial inflammatory chemokines such as monocyte chemotactic protein-1 (MCP-1, also known as CCL2). Additionally, the high expression of the surface molecule MHCII contributes to their strong antigen presentation ability. M2 macrophages are induced by IL-4 or IL-10. Unlike M1 macrophages, these cells play a role in tissue repair and inflammation suppression by producing anti-inflammatory cytokines such as IL-10 and IL-1Ra. M2 macrophages express the scavenger receptor CD163 and mannose receptor CD206 and have higher levels of CD14 than M1 macrophages (5). The distribution and transformation of M1 and M2 macrophages play a vital role in the precise regulation of inflammation (**Figure 2**). The detailed characteristics of M1 and M2 polarization are shown in **Table 1** (5, 18, 19).

**Table 1:** Characteristics of M1 and M2 macrophages.

	M1 macrophage	M2 macrophage
iNOS expression	+	–
MHCII expression	↑	↓
CD14	↓	↑
CD16/32	↑	↓
CD80	+	–
CD86	+	–
CD11c	+	–
CD163	–	+
CD206	–	+
CD209	–	+
TLR	TLR-2, TLR-4	TLR1, TLR-8
Stimulus	IFN-γ, LPS	IL-4, IL-13
Cytokines production	IL-1, IL-6, IL-12, TNF-α, CXCL9, CXCL10, CCL2	IL-4, IL-10, IL-13, IL-1Ra, TGF-β1, CCL17, CCL18, CXCL13, VEGF

“+” present. “-” absent. ↑high expression. ↓low expression.

IL, Interleukin; TLR, Toll-like receptors; TNF, Tumor Necrosis Factor; TGF, Transforming growth factor; LPS, Lipopolysaccharide; IFN, Interferon; CCL, Chemokine (C-C motif) ligand; VEGF, Vascular endothelial growth factor; CXCL, Chemokine (C-X-C motif) ligand.

To investigate how macrophage polarization affects the progression of OA, van den Bosch MH used microarray analysis and immunohistochemistry (immunostaining for CD68 and CD163) and showed that compared to the normal synovium, the OA synovium overexpressed M1-like macrophage markers such as CD86 and M2-like macrophage markers such as CD206, IL-10, and IL-1Ra. Daghestani HN analyzed the concentrations of the M2 markers CD163 and CD14 in the synovial fluid (SF) of 25 OA patients and found that the concentrations of these markers were higher in OA patients. These findings indicated that M1 and M2 macrophages proliferated in the OA synovium, which might be caused by the increase in the total number of macrophages in the OA synovium. Interestingly, some studies have noted that compared with the total number of activated macrophages, the failure to transform from the M1 to M2 subtype may play a larger role in the progression of OA (4). Yarnall BW collected synovial samples from OA dogs and found that the ratio of M1-polarized cells to M2-polarized cells was higher in the OA synovium, which indicated that disordered macrophage polarization might contribute to OA initiation and progression (20). In 2018, Zhang H found that activated synovial macrophages in OA patients were mainly M1 macrophages rather than M2 macrophages. This finding suggests that the increase in M1 synovial macrophages caused by macrophage proliferation and abnormal polarization may be a crucial reason for OA exacerbation (21).

### 3.2 Modulating the M1/M2 ratio affects OA

Although two phenotypes of macrophages (M1 and M2) have been found in the OA synovium, the specific functions of these two subtypes remain unclear. Therefore, many scientists have tried to manipulate the M1/M2 ratio and determine how this change in the M1/M2 ratio affects OA and directly show the influence of macrophage polarization on OA.

In 2016, Utomo L described the direct influence of M1 and M2 macrophages on OA for the first time. Utomo L cultured OA cartilage tissue with M1 macrophages (induced by IFN-γ and TNF-α) or M2 macrophages (induced by IL-4/IL10) *in vitro*. Then, she found that M1 macrophages exacerbated cartilage inflammation by upregulating the expression of the proinflammatory factors IL1β, IL6, MMP13 and a disintegrin and metalloproteinase with thrombospondin motif-5 (ADAMTS5). M1 macrophages also suppressed the release of aggrecan (ACAN) and collagen type II (COL2A1) and stimulated cartilage cells to produce the inflammatory mediators nitric oxide (NO) and glycosaminoglycan (GAG). On the other hand, M2 macrophages (induced by IL-10) promoted the expression of IL-1β and suppressor of cytokine signaling-1 (SOCS1) (22).

Several reports have shown that mammalian target of rapamycin (mTORC1) can promote M1 polarization and reduce M2 polarization. Deleting the tuberous sclerosis complex 1 (TSC1) gene can constitutively activate mTORC1. In contrast, deleting the Ras homolog enriched in brain 1 (Rheb1) gene inhibits the function of mTORC1. Based on these findings, Zhang H used mice with Tsc1 or Rheb1 deletion to generate collagenase-induced OA and examined the role of macrophages and their polarization in OA. He found that TSC1-knockout mice had upregulated M1-like macrophage markers and exacerbated cartilage degeneration and osteophyte formation in experimental OA. Conversely, Rheb1-knockout mice exhibited downregulated M1-like macrophage markers, upregulated M2-like macrophage markers and attenuated experimental OA. This result proves that M1 macrophages promote synovial hyperplasia, synovial inflammation and OA progression, while M2 macrophages alleviate OA progression (21). As for the specific mechanism of how M1 or M2 macrophages affect OA, scientists found M1 macrophage play its pro-inflammatory role mainly through secret cytokines like IL-1 and TNF-α, which will be explained in detail in the following passage. However, studies concerning the corresponding pathways of M2 macrophage in OA remains insufficient. Some articles reported M2 may exert protective effects through its exosomes which mainly mediated by the PI3K/AKT/mTOR signal pathway (23).

### 3.3 Various factors activate M1/M2 polarization through JAK/STAT, NFκB/MAPK, STAT6 and ROS/NLRP3 pathways in OA

Because of the importance of macrophage polarization in OA progression, scientists have tried to determine the intrinsic factors that lead to the abnormal polarization of macrophages in OA patients. Studies showed that IFN-γ and TNF-α activate the JAK/STAT pathway, whereas LPS activates the NFκB and MAPK pathways in M1 polarization. IL4/IL13 activate STAT6 and regulate transcription factors, including IRF4, PPARγ, and KLF4 in M2 polarization. Other pathways including that from JNK, PI3K/Akt, Notch, TGF-β, and hypoxia-dependent intracellular pathways have been shown to be involved in the balance of M1/M2 polarization (4). In the pathogenesis and progression of OA, several regulatory mechanisms of macrophage polarization were also discovered. Wei Z et al. found that anti-citrullinated protein antibodies (ACPAs, an autoimmune antibody) could induce M1 polarization by upregulating the expression of interferon regulatory factor 5 (IRF5) in macrophages. However, this effect was more obvious in rheumatoid arthritis (RA) than in OA because autoimmune factors are a major cause of RA but not OA (24). Another possible factor resulting in M1 polarization in OA is soluble collagens. Under disease conditions, soluble degraded forms of collagens can be detected in synovial fluids. These soluble collagens act as self-antigens and can increase M1 polarization during OA pathogenesis. Fortunately, Pal S et al. showed that sulforaphane could block M1 macrophage polarization induced by soluble collagen and convert M1 macrophages into the M2 subtype (25). Recently, some new articles reported more pathways related to M1/M2 polarization in OA patients. It was found that inhibition of TRPV4 (transient receptor potential channel subfamily V member 4), an ion channel related to oxidative stress and inflammation, delays OA progression by inhibiting M1 synovial macrophage polarization through the ROS/NLRP3 pathway (26). Another research found that MAGL (Monoacylglycerol lipase) could regulates synovial macrophage polarization vis inhibition of mitophagy. MAGL inhibition enhanced the mitophagy levels of M1 macrophages (27). However, the mechanism involved in regulating macrophage polarization by MAGL requires further study. Collectively, those studies showed the possible intrinsic factors that cause an imbalance in macrophage polarization in OA. Further studies are needed to clarify the detailed mechanisms of abnormal polarization states.

## 4 Synovial macrophages help distinguish the two OA subgroups

Although the discovery of M1 and M2 polarization in synovial macrophages was a huge breakthrough in understanding the pathogenesis of OA, this is not the only factor in the initiation and progression of this disease because many intermediate stage macrophages are present in the OA synovium despite these two extreme subtypes (18). To better understand the heterogeneity of this disease, scientists further classified OA based on the gene expression of total synovial macrophages. It was recently reported that knee OA can be divided into 2 subgroups based on the properties of activated synovial macrophages. After RNA sequencing of OA and inflammatory-arthritis (IA) synovial tissue macrophages, Wood MJ identified 2 distinct OA subgroups based on the hierarchical heatmap of the top 500 most variable genes. One OA group was distinct, and another OA group was more similar to IA. The distinct OA group was termed classical OA (cOA), and the IA-like OA group was termed inflammatory-like OA (iOA). Subsequently, synovial macrophage gene expression analysis and gene set enrichment analysis were performed to determine whether the two OA subtypes had different disease mechanisms. The results demonstrated that the iOA group overexpressed cell cycle-related genes such as MKI67 (the proliferation marker Ki67–encoding gene) compared to the cOA group. Consistent with this finding, flow cytometry proved that the synovial tissue of the iOA group contained a higher ratio of macrophages than that of the cOA group. In general, the heterogeneous gene expression signatures of OA synovial macrophages may represent different cellular disease mechanisms and be an important consideration in OA classification (18).

## 5 Synovial macrophages exacerbate OA by producing proinflammatory cytokines, affecting synovial fibroblasts and interacting with chondrocytes

### 5.1 OA synovial macrophages stimulate proinflammatory cytokines

Takano S found that synovial macrophages highly expressed TNF-α and IL-1 (14). Consistent with this finding, Bondeson J collected the supernatant of macrophage-depleted OA synovial tissue culture and found that the concentrations of IL1 and TNF-α declined (8). Zhang H performed mRNA sequencing on cultured macrophages from TSC1-knockout mice and found that the cytokines IL-1, IL-6 and TNF-α were upregulated. In addition to the two most well-known macrophage-derived cytokines (TNF-α and IL-1), many other cytokines and chemokines are directly or indirectly involved in macrophage-mediated OA progression. Zhang H found that R-spondin-2 (Rspo2) played an important role in OA. Interarticular injection of Rspo2 exacerbates OA, and the injection of Rspo2 antibodies reversed the exacerbation (21). Synovial macrophages can directly or indirectly induce the production of matrix-degrading enzymes such as MMPs by chondrocytes and synovial fibroblasts (28). Moreover, Takano S discovered that synoviocytes could produce NGF and cause pain in OA (14). Haywood L found that synovial macrophages secreted VEGF to exacerbate synovial angiogenesis (7). Another interesting cytokine is TGF-β. Synovial macrophages can induce osteophyte formation in OA *via* the production of TGF-β, BMP-2, and BMP-4 (13). Chemokines that can mediate macrophage accumulation are also crucial participants in synovitis. Synovial macrophages not only produce chemokines such as CCL2 but also stimulate other tissue cells to secrete chemokines that further enhance macrophage accumulation (5, 29).

### 5.2 OA synovial macrophages interact with synovial fibroblasts

**Figure 3:**
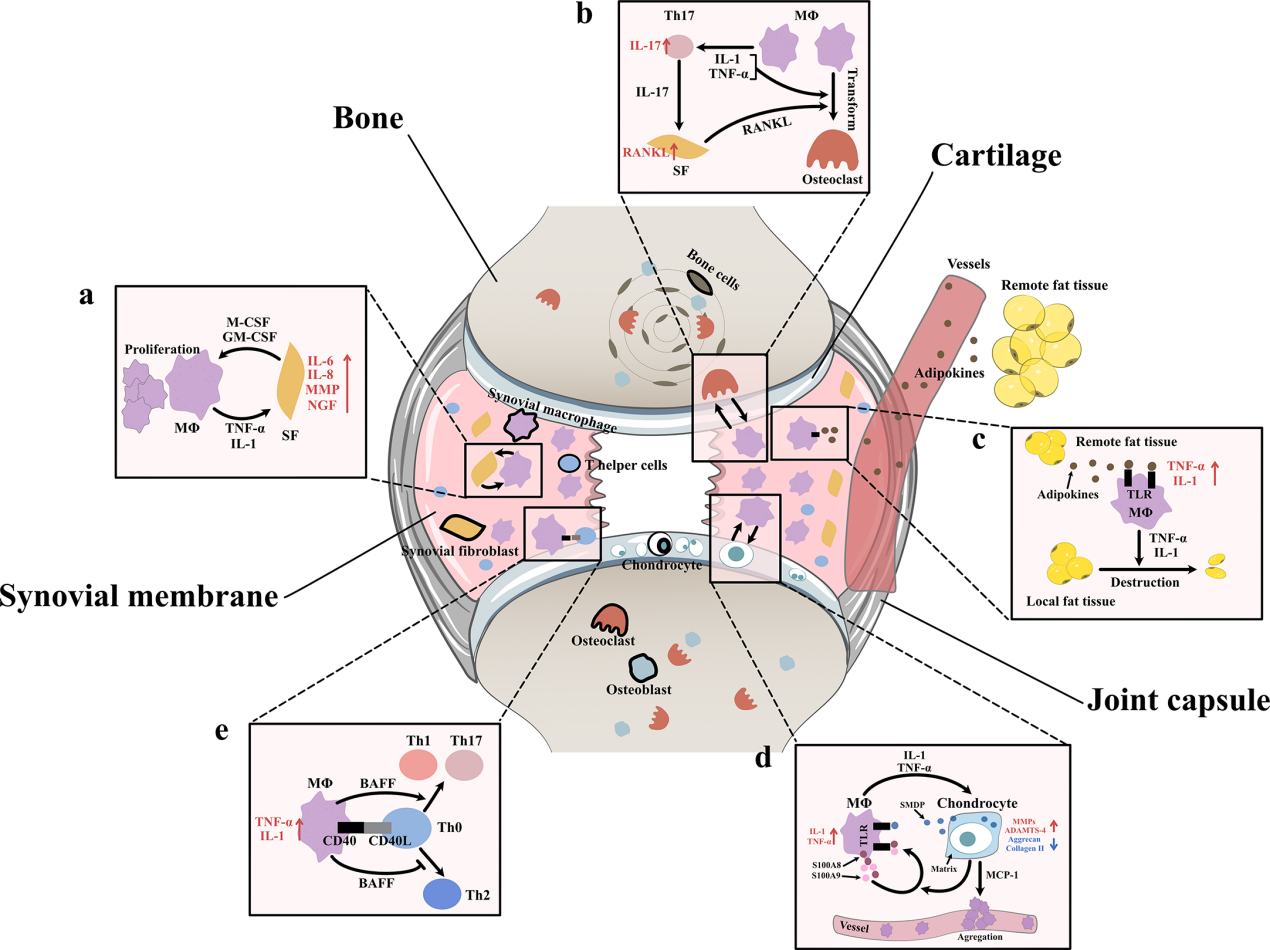
Interaction between synovial macrophages and other cells **(A)** Synovial macrophages and synovial fibroblasts: Synovial macrophages release interleukin (IL)-1, tumor necrosis factor-α (TNF-α) to increase the expression of IL-6, IL-8, matrix metalloproteinases (MMPs), nerve growth factor (NGF) in synovial fibroblasts. Fibroblasts stimulate macrophage proliferation by producing macrophage colony stimulating factor (M-CSF) and granulocyte-macrophage colony stimulating factor (GM-CSF) **(B)** Synovial macrophages and osteoclast: Macrophages can transform into osteoclast when stimulated by IL-1, TNF-α, and especially receptor activator of nuclear factor κB ligand (RANKL). It is found synovial macrophages indirectly elevate RANKL level through CD4+ T cells and synovial fibroblast cells. Macrophages secret TNF-α and IL-1 to stimulate the differentiation of Th17 cells, which subsequently release IL-17 to induce the RANKL production of synovial fibroblasts. **(C)** Synovial macrophage and adipocytes: Fat tissues outside the joint secret adipokines like leptin, adiponectin and visfatin into blood circulation. Then adipokines get into synovial membrane and activate macrophage TLR to increase TNF-α, IL-1 expression. These macrophage-derived cytokines, in turn, lead to destruction and remodeling of local joint adipose tissue, which is a protective factor of osteoarthrosis. **(D)** Synovial macrophages and chondrocytes: Synovial macrophages release IL1, TNF-α to upregulate the expression of MMPs, A Disintegrin and Metalloproteinase with Thrombospondin-4 (ADAMTS-4) while downregulate Aggrecan and collagen II. In turn, injured cartilage produces soluble matrix degradation products (SMDP) and monocyte chemotactic protein-1 (MCP-1). It also leads to increased levels of two autocrine proteins S100A8 and S100A9 in macrophages. Both SMDP and S100A8/S100A9 function through activating Toll-like receptor (TLR) expressed on macrophages. MCP-1 functions by promoting macrophage aggregation. **(E)** Synovial macrophages and CD4+T cells: Synovial macrophages secret B cell Activating Factor (BAFF) to promote pro-inflammatory Th1 cells and Th17 cells while suppress anti-inflammatory Th2 response. Meanwhile, CD4+T cells also contribute to macrophage activation through CD40/CD40L co-stimulatory pathways.

After Bondeson J removed macrophages from OA synovial cultures, the macrophage-derived cytokines IL1 and TNF-α decreased, and the fibroblast-derived cytokines IL-6, IL-8, and MMPs were also reduced (8). In addition, after stimulating synovial fibroblasts with TNF-α and IL1, Takano S observed a strong increase in NGF (related to OA pain) (14). *In vitro* coculture experiments indicated that macrophages triggered the emergence of proinflammatory fibroblasts that expressed not only the typical fibroblast-derived proinflammatory mediator IL-6 but also mediators of cartilage degradation, such as MMPs. These cells could also produce collagens that exacerbate synovial fibrosis. The activation of functional synovial fibroblasts also impacts the function and phenotype of macrophages. Synovial fibroblasts send attraction signals to macrophages through the mechanical movement of ECM fibers caused by the contraction of fibroblasts. Moreover, fibroblasts have also been shown to produce macrophage colony stimulating factor (M-CSF) and granulocyte-macrophage colony stimulating factor (GM-CSF), two conventional mediators that stimulate the mononuclear phagocyte system (30) (**Figure 3A**).

### 5.3 OA synovial macrophages interact with chondrocytes

Aberrant hyperplasia and differentiation of chondrocytes are crucial for OA initiation and progression. It has been reported that the severity of synovitis is closely related to the severity of joint dysfunction due to the interaction between cartilage and the synovium (3). MMPs are a group of matrix-degrading enzymes that mediate cartilage erosion, and several of them have been suggested to participate in OA. VDIPEN, an MMP-generated neoepitope, is positively associated with cartilage breakdown. In 2007, synovial macrophages were removed using clodronate-laden liposomes prior to the creation of a mouse OA model, and Blom AB proved that synovial macrophages affected cartilage function through MMPs. He found that the expression of MMP-3 and MMP-9 in synovial tissue but not cartilage tissue was significantly decreased in macrophage-depleted mice, indicating that synovial macrophages could directly affect cartilage by producing MMPs. A dramatic decrease in the expression of VDIPEN was also noticed (28). In 2018, Zhang H found that the expression of the chondrogenesis regulator gene sry-box transcription factor 9 (*Sox*9) and the chondrocyte marker genes *Col2a1* and *Acan* was decreased, while the expression of *Col10a1* and runt-related transcription factor 2 (*Runx2*), which are associated with chondrocyte differentiation, was enhanced in OA mice with increased M1 polarization. A high level of cartilage matrix mineralization was also observed, indicating that activated synovial macrophages could promote abnormal chondrocyte maturation and differentiation through cytokines/enzymes (21). It was later discovered that activated synovial macrophages could secrete TNF-α and IL-1 to increase cartilage expression of proinflammatory cytokines such as MMPs and ADAMTS-4 and decrease protein levels of ACAN and collagen II (31–33). Another newly-discovered mediator of this macrophage–cartilage communication was extracellular vehicles (EVs). EVs are cell-derived membrane vesicles containing numerous types of bioactive molecules, including proteins, lipids, mRNAs, microRNAs and DNA. In an *in vitro* study, scientists found when EVs secreted by M1 reached chondrocytes, they transferred their cargos into chondrocytes and caused significant increase in the expression of IL-6, MMP13, and ADAMTS5 which further led to cartilage degeneration (34). The stimulation effect between cells is mutual. Synovial macrophages also expressed increased IL-1β and VEGF when cultured with abnormal cartilage cells from OA joints (33). Further study showed that the underlying mechanism of the proinflammatory effect of injured cartilage may be associated with SMDPs and MCP-1 (also known as CCL2). When articular cartilage is injured, molecular fragments of cartilage, such as SMDPs, irritate the synovium and bind with TLRs expressed on synovial macrophages to increase the expression of multiple proinflammatory cytokines that further exacerbate cartilage injury (35–37). MCP-1 is an important leukocyte chemotactic factor that is expressed by injured cartilage cells and can promote macrophage accumulation (29, 38). Damage to the cartilage could also lead to increased levels of the alarmins S100A8 and S100A9, two autocrine proteins produced by synovial macrophages that act on themselves to induce proinflammatory cytokines such as IL and TNF through TLRs (39). These results indicated the mutual effects of activated synovial macrophages and OA cartilage (**Figure 3D**).

### 5.4 OA synovial macrophages interact with T cells

As mentioned before, another important change in the OA synovium is the increase in CD4+ T cells. Instead of acting alone, these lymphocytes interact with synovial macrophages in multiple ways. For instance, the level of B-cell activating factor (BAFF), which is a cytokine produced mainly by macrophages, is elevated in OA synovial fluid (40). As a member of the TNF family, BAFF can not only support the survival and proliferation of B cells but also play a role in T-cell activation. BAFF can promote proinflammatory Th1 cells and Th17 cells while suppressing the anti-inflammatory Th2 response, which may serve as evidence of the interplay between macrophages and T cells (41). When macrophages trigger a specific immune response, T cells also contribute to macrophage activation through the CD40/CD40L costimulatory pathways. CD40 is a member of the TNF receptor family that is mainly expressed on the surface of antigen-presenting cells such as macrophages, and its ligand CD40L is almost exclusively expressed by activated CD4^+^ T cells. When CD40 and CD40L interact, it leads to macrophage activation and B-cell differentiation (42, 43). In turn, activated macrophages further enhance cellular and humoral immune responses, which result in proinflammatory positive feedback. CD40L mRNA levels are elevated in the synovium during the early onset of OA (40) (**Figure 3E**).

### 5.5 OA synovial macrophages cause bone destruction through osteoclastogenesis

As specialized phagocytes, osteoclasts play an important role in the metabolic balance of bone tissue. An equivalent level of osteoclasts and osteoblasts is a key factor in healthy bone remodeling. However, in OA joints, osteoclasts are aberrantly activated, which leads to typical subchondral bone destruction and absorption (44). Notably, this activation of osteoclasts may involve synovial macrophages. CD14+ macrophages extracted from the synovial fluid of OA patients can transform into functional osteoclasts when stimulated by receptor activator of nuclear factor κB ligand (RANKL, an osteoclast differentiation factor), IL-1α and TNF-α (44). In addition to direct transformation, macrophages can also indirectly promote osteoclast activation through CD4+ T cells and synovial fibroblasts. In rheumatoid arthritis (RA), cytokines such as TNF-α and IL-1 secreted by macrophages stimulate the differentiation of Th17 cells, which subsequently release IL-17 to induce RANKL production in synovial fibroblasts (45). In OA, osteoclastogenesis induced by CD4+ T cells has also been discovered; however, whether the mechanism of this change is identical to that in RA has not yet been clarified (46) (**Figure 3B**).

### 5.6 OA synovial macrophages interact with adipocytes

Obesity is not only a risk factor for OA occurrence but also an exacerbating factor in OA development. Animal experiments have shown that a high-fat diet (HFD) can exacerbate synovitis in OA mice, which is characterized by increased infiltration of synovial macrophages and partial loss of joint adipose tissue (47). It is assumed that fat tissues outside the joint rather than those inside the joint are responsible for the exacerbation of synovitis. Fat tissues outside the joint may secrete adipokines such as leptin, adiponectin and visfatin to remotely control the number and function of macrophages in the synovium *via* blood circulation. Markedly elevated blood adipokines and an increased number of synovial macrophages and TNF-α and IL-1 expression were found in HFD-fed OA mice (47). Among adipokines, visfatin was recently shown to be capable of inducing inflammatory responses by activating Toll-like receptor 4 (TLR4) expressed on monocytes and macrophages and subsequently inducing the secretion of TNF and IL1 (48, 49). Leptin and adiponectin are also closely related to the nonspecific immune response (50). Local fat tissue in OA joints, on the other hand, is thought to protect against OA progression. A study of OA synoviocytes cultured with microfragmented adipose tissue (MF) showed that MF reduced the release of CCL2 and MMPs, downregulated TLR4 and increased tissue inhibitor of metalloproteinases (TIMP-1, an MMP-9 inhibitor) in synoviocytes (51). Another clinical study of 977 OA patients showed that the infrapatellar fat pad (a part of local fat tissue) is beneficially associated with radiographic OA, MRI structural pathology and knee pain (52). It is worth mentioning that the relationship between macrophages and adipocytes is mutual. Macrophage-derived proinflammatory cytokines contribute to the destruction and remodeling of local joint adipose tissue (47) (**Figure 3C**).

## 6 Drugs targeting macrophages and macrophage-associated inflammatory pathways

### 6.1 Drugs targeting macrophage-associated inflammatory cytokines (TNF-α, IL-1)

Recent studies have shown that the IL-1 receptor antagonist (IL-1Ra) anakinra exerts positive effects on articular inflammatory diseases such as rheumatoid arthritis and systemic juvenile idiopathic arthritis (53). In some animal experiments, it has been proven that IL-1Ra can prevent the progression of experimental OA by inhibiting the generation and activity of IL-1 (54, 55). However, different conclusions were drawn by two RCTs in which no significant clinical improvements in OA were detected after interarticular injection of the IL-1Ra anakinra or the IL-1 receptor antibody AMG108 (56, 57) (**Table 2**). Overall, the mechanism and clinical application of IL-1Ra in OA treatment require further research.

**Table 2:** Characteristics of studies researching macrophage-targeting drugs for OA treatment.

First Author, Year	Study Type	Country	Population	Experimental environment	Interference
Drugs targeting macrophage-derived inflammatory cytokines					
*IL-1 antagonist*					
Pelletier JP, 1997^54^	Animal study	Canada	17 dogs	*In vivo* + *in vitro*	Surgery induced knee OA + intra-articular injection of autologous cells transduced with the IL-1Ra gene
Caron JP, 1996^55^	Animal study	Canada	16 dogs	*In vivo*	Surgery induced knee OA + intra-articular injection of recombinant human IL-1Ra
Chevalier X, 2009^56^	RCT	USA	170 knee OA patients	*In vivo*	Anakinra (an IL-1Ra) intra-articular injection
Cohen SB, 2011^57^	RCT	USA	228 knee OA patients	*In vivo*	AMG 108 (a fully human monoclonal antibody to IL-1R) subcutaneously or intravenously injection
*TNF blockade*					
Grunke M, 2006^58^	Case report	Germany	a 68 year old male knee OA patient	*In vivo*	Adalimumab (human TNF antibody) subcutaneously injection
Magnano MD, 2007^59^	Pilot trial	USA	12 hands OA patients	*In vivo*	Adalimumab intra-articular injection
Fioravanti A, 2009^60^	Pilot trial	Italy	10 hands OA patients	*In vivo*	Infliximab (TNF-α antagonist) intra-articular injections
Chevalier X, 2015^61^	RCT	France	99 hands OA patients	*In vivo*	Adalimumab subcutaneous injections
Aitken D, 2018^62^	RCT	Australia	51 hands OA patients	*In vivo*	Adalimumab subcutaneous injections every other week
Drugs inducing M2 polarization					
Dai M, 2018^63^	Animal study	China	Rats	*In vivo* + *in vitro*	Surgery-induced knee OA+ SCII intra-articular injections
Shu CC, 2020^64^	Animal study	Australia	Mice	*In vivo*	Surgery induced knee OA+ intra-articular injection of Hymovis (a hyaluronan hexadecylamide derivative)
Manferdini C, 2017^65^	In vitro test	Italy	12 human OA synovial tissues	*In vitro*	M1 macrophages from OA tissues were co-cultured with ASC
Cherian JJ, 2015^66^	RCT	USA	102 knee OA patients	*In vivo*	GEC-TGF-β1 intra-articular injection
Ha CW, 2015^67^	RCT	South Korea	27 knee OA patients	*In vivo*	GEC-TGF-β1 intra-articular injection (high dose)
Cho JJ, 2017^68^	RCT	South Korea	27 knee OA patients	*In vivo*	GEC-TGF-β1 intra-articular injection (high dose)
Cho J, 2016^69^	RCT	South Korea	156 knee OA patients	*In vivo*	Invossa™ intra-articular injection
Lee H; 2018^70^	Animal study	South Korea	Rats	*In vivo*	Surgery-induced knee OA+ Invossa™ intra-articular injection
Drugs inhibiting M1 polarization					
Wang H, 2021^71^	Animal study	China	Mice	*In vivo*	Collagenase-induced OA+ Frugoside intra-articular injection

First Author, Year	Drug Name	Outcome	Result	Mechanism
Drugs targeting macrophage-derived inflammatory cytokines				
*IL-1 antagonist*				
Pelletier JP, 1997^54^	Autologous cells transduced with the IL-1Ra gene	Macroscopic and microscopic changes	IL-1Ra suppressed early experimental osteoarthritis	Block IL-1 receptor
Caron JP, 1996^55^	Recombinant human IL-1Ra	Macroscopic and microscopic changes; RNA expression	IL-1Ra suppressed experimental osteoarthritis	Block IL-1 receptor
Chevalier X, 2009^56^	Anakinra	WOMAC score; Adverse events	Anakinra didn’t improve joint pain and function	Block IL-1 receptor
Cohen SB, 2011^57^	AMG 108	WOMAC score; Adverse events	AMG 108 didn’t improve joint pain and function	Block IL-1 receptor
*TNF blockade*				
Grunke M, 2006^54^	Adalimumab	Self-reported joint function and pain; MRI	Adalimumab improved joint function and pain	Bind with TNF
Magnano MD, 2007^59^	Adalimumab	ACR response; Adverse events	Adalimumab leaded to pain relief and radiographic alleviation	Bind with TNF
Fioravanti A, 2009^60^	Infliximab	Radiographs changes; VAS pain score	Infliximab leaded to pain relief and radiographic alleviation	Bind with TNF
Chevalier X, 2015^61^	Adalimumab	Number of people with 50% of VAS improvement;	Adalimumab didn’t improve joint pain and function	Bind with TNF
Aitken D, 2018^62^	Adalimumab	VAS pain score; AUSCAN pain score; MRI	Adalimumab didn’t improve joint pain and function	Bind with TNF
Drugs inducing M2 polarization				
Dai M, 2018^63^	SCII	Macroscopic and microscopic changes; RNA expression	SCII significantly improved the structural integrity of the cartilage tissue in OA rats	SCII increases M2-polariation of macrophages; SCII inhibites chondrocyte apoptosis
Shu CC, 2020^64^	Hymovis	Macroscopic and microscopic changes; RNA expression	Hymovis reduces allodynia at an early but not late stage of OA	Hymovis increased M2- macrophages.
Manferdini C, 2017^65^	ASC	Microscopic changes; RNA expression	M1-like macrophage markers downregulated while M2-like macrophage markers upregulated when cultured with ASC	ASC activates M2-macrophages while inhibits M1-macrophages
Cherian JJ, 2015^66^	GEC-TGF-β1	IKDC score; VAS pain; Adverse events	GEC-TGF-β1 patients had improved scores	TGF-β induces osteogenesis and chondrogenesis; TGF-β increase M2 polarization
Ha CW, 2015^67^	GEC-TGF-β1	IKDC score; WOMAC score; VAS pain;	GEC-TGF-β1 patients had improved scores	TGF-β induces osteogenesis and chondrogenesis; TGF-β increase M2 polarization
Cho JJ, 2017^68^	GEC-TGF-β1	MRI	GEC-TGF-β1 patients had improved MRI assessment parameters	TGF-β induces osteogenesis and chondrogenesis; TGF-β increase M2 polarization
Cho J, 2016^69^	Invossa™	IKDC score; WOMAC score; VAS pain; KOOS score	Invossa™ patients had improved scores	TGF-β induces osteogenesis and chondrogenesis; TGF-β increase M2 polarization
Lee H; 2018^70^	Invossa™	Pain behavior; Histological staining; RNA expression	Invossa™ rats showed pain relief, cartilage regeneration. It encouraged M2 polarization and inhibit M1 polarization	TGF-β induces osteogenesis and chondrogenesis; TGF-β increase M2 polarization
Drugs inhibiting M1 polarization				
Wang H, 2021^71^	Frugoside	Histological staining; RNA expression; Protein level;	Frugoside attenuates synovial inflammation and delays the development of OA	Frugoside inhibits macrophage M1 polarization

RCT, randomized control trial; OA, osteoarthrosis; IL-1Ra, Interleukin-1 receptor antagonis; WOMAC, Western Ontario and McMaster Universities Osteoarthritis Index; ACR, American College of Rheumatology; VAS, Visual Analogue Scale; AUSCAN, Australian/Canadian Hand OA Index; SCII, squid type II collagen; ASC, adipose mesenchymal stromal cell; GEC-TGF-β1, genetically engineered chondrocytes virally transduced with TGF-β1; IKDC, International Knee Documentation Committee; KOOS, Knee Injury and Osteoarthritis Outcome Score.

TNF-α is a crucial proinflammatory mediator in the initiation of OA. Therefore, anti-TNF therapy has become a hot spot of research in recent years. In 2006, Grunk M reported that a 68-year-old male patient with bilateral OA of the knees gained remarkable pain relief after subcutaneous injection of the TNF antibody adalimumab. This case report indicated exciting medical efficacy of TNF antibody on OA human beings rather than animals for the first time (58). Subsequently, Magnano MD carried out a pilot study in which 12 patients with hand OA were treated with adalimumab (40 mg/2 w) for 12 weeks. The treatment group had statistically significant improvements in the number of swollen joints and improvements in the number of tender joints, grip strength, disability and pain (59). In another pilot study, 10 women with bilateral OA of the hand were enrolled. The participants were treated with interarticular injections of the TNF antibody infliximab in one hand and an equal amount of saline in the other hand. After 12 months of treatment, the hands treated with infliximab exhibited more significant pain relief and radiographic alleviation than those treated with saline (60). Although these case reports and pilot studies demonstrated that TNF antibodies could prevent the progression of OA, several RCTs yielded opposite conclusions. A randomized controlled crossover trial enrolled 43 patients with erosive hand OA and treated them with adalimumab or saline. Another RCT enrolled 85 hand OA participants and treated them with adalimumab or saline. Both RCTs showed similar pain scores and radiographic scores between the groups (61, 62).

### 6.2 Drugs that induce M2 polarization

Since M2 synovial macrophages play an anti-inflammatory role in OA, drugs that induce M2 polarization might be a promising strategy for the treatment of OA. In this section, we will summarize several drugs that increase M2 macrophages.

Dai M found that squid type II collagen (SCII) could increase the production of collagen type II and GAG and promote cartilage repair *in vitro* by increasing the ratio of M2 macrophages and the levels of chondrogenic cytokines (TGF-β1 and TGF-β3) in synovial fluid. Additionally, the glycine in SCII could activate the glycine receptor on chondrocytes to decrease intracellular calcium concentrations, resulting in the inhibition of chondrocyte apoptosis. *In vivo* experiments using a rat model of OA also proved that SCII could induce M2 polarization, increase chondrogenic cytokines and inhibit chondrocyte apoptosis and MMP13 production (63).

Shu CC found that OA mice treated with Hymovis^®^ (a hyaluronan derivative) showed significantly higher ratios of M2 macrophages. This may explain the longer-term effect of Hymovis^®^ on pain relief and the decrease in joint cystic fibrosis (64).

Manferdini C found that adipose mesenchymal stromal cells (ASCs) could transform M1 macrophages into M2 macrophages through PGE2. In cocultures containing ASCs and M1 macrophages, the M1 macrophage factors IL1β, TNFα, IL6, MIP1α/CCL3, S100A8 and S100A9 were downregulated, while the M2 markers IL10, CD163 and CD206 were upregulated. This change could be blocked by a PGE2 receptor antagonist. This result indicates that ASCs can be a promising OA treatment (65).

TGF-β may have anti-inflammatory and immunosuppressive properties. The cytokine TGF-β can induce osteogenesis and chondrogenesis and plays a role in cell growth, differentiation and extracellular matrix protein synthesis. In addition, TGF-β can promote proteoglycan synthesis and chondrocyte proliferation. Multiple clinical trials have proven that interarticular injection of genetically engineered allogeneic human chondrocytes expressing TGF-β1 (GEC-TGF-β1) can alleviate pain and articular deterioration in OA patients (66–68). Based on the efficacy of TGF-β, INVOSSA-K was discovered. As a novel cell and gene therapy for OA, INVOSSA-K contains nontransformed human chondrocytes and GEC-TGF-β1. In a phase III clinical trial, the pain scores and function scores of OA patients significantly improved at the 1-year follow-up after a single injection of Invossa™ (69). To investigate how Invossa™ alleviates OA, Lee H injected Invossa™ into a rat OA model. Using RT-PCR and histological staining, he found that INVOSSA-K increased the number of M2 macrophages and decreased the number of M1 macrophages, indicating that INVOSSA-K might play an anti-inflammatory role by manipulating macrophage polarization (70). Though from a scientific point of view, the principals of INVOSSA-K treatment is interesting, it is still need to note that this treatment is not available to most western countries yet because the manufacture mislabeled the ingredients used (**Table 2**).

### 6.3 Drugs that inhibit M1 polarization

Frugoside (FGS) is isolated from *Calotropis gigantea* and possesses a special cardenolide structure. It was recently discovered that cardiac glycosides are beneficial for the cardiovascular system and can alleviate inflammatory symptoms. In 2021, Wang H found that FGS could delay the development of OA by inhibiting the M1 polarization of synovial macrophages. The expression of iNOS, F4/80, Col2α1, MMP13 and M1 macrophage factors was analyzed by RT-PCR, WB, flow cytometry and immunofluorescence staining after injecting FGS into the damaged joints of collagenase-induced OA (CIOA) mice. The results demonstrated that FGS inhibited M1 macrophage polarization, which subsequently decreased the secretion of IL‐6 and TNF‐α. Further results showed that FGS could inhibit M1 macrophage polarization by partially downregulating the expression of miR-155 (71) (**Table 2**).

## 7 Conclusions

Our review summarizes the role of synovial macrophages in the onset and progression of OA. Current evidence indicates that OA synovitis mainly arises from the proliferation and activation of synovial macrophages. The accumulation of M1 macrophages and imbalanced M1/M2 ratio promote OA development. M1 macrophages exacerbate inflammation by secreting multiple proinflammatory cytokines and activate surrounding tissue cells. The crosstalk between macrophages and these surrounding cells is summarized in this review. Drugs targeting macrophages and macrophage-associated inflammatory pathways are also summerized in this review. IL-1 receptor antagonists and TNF-α receptor antagonists might be able to ameliorate OA. New therapies such as SCII, Hymovis^®^, ASC, INVOSSA and Frugoside can either enhance M2 polarization or inhibit M1 polarization, which subsequently reduces the inflammatory response in OA patients.

## Author contributions

KZ and JQR contributed equally as co-first author. All authors contributed to the article and approved the submitted version.
